# Correction: Pharmacotherapy of major depressive disorder in older adults: from an evidence-informed stepwise algorithm to precision medicine

**DOI:** 10.1038/s41386-026-02387-1

**Published:** 2026-03-18

**Authors:** Eric J. Lenze, Jordan F. Karp, Marie Anne Gebara, Ginger E. Nicol, Benoit H. Mulsant

**Affiliations:** 1 Department of Psychiatry, Washington University School of Medicine, St Louis, MO USA; 2 Department of Psychiatry, University of Arizona School of Medicine, Tucson, AZ USA; 3 Department of Psychiatry, University of Pittsburgh School of Medicine, Pittsburgh, PA USA; 4 Centre for Addiction and Mental Health, Department of Psychiatry, Temerty Faculty of Medicine, University of Toronto School of Medicine, Toronto, ON Canada; 5 The Royal Ottawa Hospital, Ottawa, ON Canada

**Keywords:** Depression, Outcomes research

Correction to: *Neuropsychopharmacology* 10.1038/s41386-026-02338-w, published online 19 February 2026

Figure 3’s legend is updated. The legend said “b The “Benign vs. pernicious depression” approach proposes that…; whereas the figure itself shows “Simple vs. complex depression” (and elsewhere in the paper we use that terminology). Therefore, the legend should say “b The “Simple vs. complex depression” approach proposes that...

The section entitled “Part 2 conclusion” refers to Figure 3 – twice. Both times, it should say Figure 2.

And the section entitled “Precision medicine approach 2: targeting brain aging and biological aging” refers to Figure 2 – it should say Figure 1. That section also says “(because of the longevity era 3)” and it should just say “(because of the longevity era)”

The original article has been corrected.

